# Supplementary material for: Public awareness of gastric cancer risk factors and screening behaviours in Shijiazhuang, China: A community-based survey
Source: PLoS One. 2024 Oct 7;19(10):e0311491. doi: 10.1371/journal.pone.0311491 (PMC11458051; doi:10.1371/journal.pone.0311491)
Supplement: Attachment — Submitted filename: Response to Reviewer 2.docx [file pone.0311491.s002.docx]

Dear Reviewer #2,

Thank you very much for your positive feedback and valuable suggestions on our manuscript titled "Public Awareness of Gastric Cancer Risk Factors and Screening Behaviours in Shijiazhuang, China: A Community-based Survey." We greatly appreciate your time and effort in reviewing our work. We are especially grateful for your kind words regarding the quality and importance of our study to the scientific community.

We have carefully considered your suggestions and made the following revisions to improve the manuscript. In the revised manuscript, all the changes have been highlighted in yellow.

**Comment 1:**

Present an international overview of the theme in the introduction (what was done similarly in other countries, or even what was not done)

**Response:**

We have expanded the **Introduction** to provide an international perspective on the topic. Please see **Page 4 , lines 77-79, Page4-5, lines 85-92, and Page 5-6, lines 102-112**.

**Comment 2:**

Improve the practical implications of the study (How important is the study for society? How important is the study for professionals? How important is the study for the health system? Etc..

**Response:**

1 We have added another paragraph in **Discussion**.

“Our findings have important clinical and public health implications. First, we provide valuable reference data to help decision-makers enhance cancer awareness and screening rates. It is advisable for them to establish an authoritative platform for science communication, to compile and disseminate core information and key points on cancer prevention and treatment. Simultaneously, they should optimize cancer screening management and establish a tiered screening system. Secondly, professionals will tailor health education initiatives to address public misconceptions and knowledge gaps, thereby tackling inequalities in awareness. Additionally, barriers to screening motivate doctors to develop novel, non-invasive, and precise biomarkers, which further advance screening technologies. Third, although raising public awareness is a relatively gentle method for achieving behavioral change, it is immensely beneficial for enhancing the overall health literacy of the population and encouraging long-term adherence to healthy behaviors. Better understanding of risk factors and increased prevention efforts offer cost-effective and sustainable means to reduce the global cancer burden in the longer term.”(**Page 21-22, Lines 426-440**)

2 We have made minor adjustments in other parts of the **Discussion** to ensure consistency in tone and completeness. Please see **Page 17, lines 339-344, and Page 21, lines 417-422**.

Once again, we sincerely appreciate your constructive feedback. Your suggestions have undoubtedly helped improve the quality and clarity of our manuscript. We look forward to your further comments and hope that our revised manuscript meets your expectations.

Thank you for your continued support.

Sincerely,

Shuping Zhao and co-authors

21 Jun., 2024

The Second Hospital of Hebei Medical University

E-mail: 315965707@qq.com
